# Inherited Variation in Vitamin D Genes and Type 1 Diabetes Predisposition

**DOI:** 10.3390/genes8040125

**Published:** 2017-04-20

**Authors:** Marissa Penna-Martinez, Klaus Badenhoop

**Affiliations:** Division of Endocrinology, Diabetes and Metabolism, Department of Medicine 1, University Hospital Frankfurt, Theodor-Stern-Kai 7, D-60590 Frankfurt am Main, Germany; Marissa.Penna-Martinez@kgu.de

**Keywords:** immune modulation, pharmacogenomics, nuclear hormone action

## Abstract

The etiology and pathophysiology of type 1 diabetes remain largely elusive with no established concepts for a causal therapy. Efforts to clarify genetic susceptibility and screening for environmental factors have identified the vitamin D system as a contributory pathway that is potentially correctable. This review aims at compiling all genetic studies addressing the vitamin D system in type 1 diabetes. Herein, association studies with case control cohorts are presented as well as family investigations with transmission tests, meta-analyses and intervention trials. Additionally, rare examples of inborn errors of vitamin D metabolism manifesting with type 1 diabetes and their immune status are discussed. We find a majority of association studies confirming a predisposing role for vitamin D receptor (VDR) polymorphisms and those of the vitamin D metabolism, particularly the CYP27B1 gene encoding the main enzyme for vitamin D activation. Associations, however, are tenuous in relation to the ethnic background of the studied populations. Intervention trials identify the specific requirements of adequate vitamin D doses to achieve vitamin D sufficiency. Preliminary evidence suggests that doses may need to be individualized in order to achieve target effects due to pharmacogenomic variation.

## 1. Introduction

The growing incidence of type 1 diabetes (T1D) is understood to result from an interplay of several factors including environment, nutrition and genetics. One of the environmental and nutritional factors may be a vitamin D deficiency that is highly prevalent and increases the risk for T1D as well as other autoimmune disorders [1,2]. In vitro studies could show a protective effect of active vitamin D for cytokine treated human pancreatic islets [3]. This field of research is of continuing interest with a steady increase in publications over recent years. Due to the lack of a causal therapy in T1D, vitamin D research intended to pave the way for novel immune modulatory concepts both for prevention as well as therapy.

Vitamin D is structurally related to the steroid hormones and its mechanism of action also involves a nuclear receptor similar to thyroid, gonadal and adrenal hormones. The physiological effects extend from the classical bone and calcium/phosphate regulation to muscle, vasculature, immunity, skin, gut and brain which explains that the vitamin D receptor (VDR) is expressed on a vast number of cells which respond to vitamin D. The immune effects of vitamin D on dendritic cells, macrophages and T lymphocytes have attracted major attention since they hold promise for novel therapies [4,5].

## 2. Vitamin D Pathways

**Figure 1:**
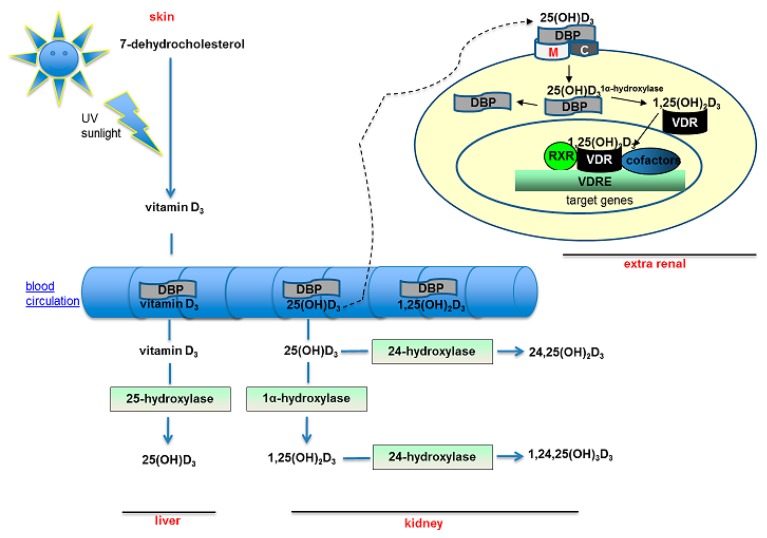
Vitamin D pathway: The vitamin D synthesis goes through a series of hydroxylation steps in which the 25-hydroxylase (CYP2R1) and 1-α-hydroxylase (CYP27B1) are involved. The resulting 25(OH)D_3_ and 1,25(OH)_2_D_3_ are transported into the circulation bound to the vitamin D binding protein (DBP). The 25(OH)D_3_ enters into the cells via the megalin/cubilin complex. Intracellularly, 1,25(OH)_2_D_3_ binds to the vitamin D receptor (VDR) and exerts its genomic effects. In this manner, vitamin D can (1) suppress PTH synthesis in parathyroid glands; (2) increase bone mineralization; (3) increase absorption of calcium and phosphate in the intestine; (4) induce the differentiation of immune cells; and (5) improve the haematopoiesis of red blood cells. Finally, the degradation of vitamin D occurs via 24-hydroxylase (CYP24A1).

There are two major forms of vitamin D: vitamin D_2_ and vitamin D_3_. While D_2_ (ergocalciferol) is of exogenous origin via food intake, vitamin D_3_ (cholecalciferol) comes primarily from skin production through photochemical reaction of precursors and thereby reflects endogenous synthesis. This is initiated upon cutaneous exposure to ultraviolet (UV) B radiation, resulting in the conversion of 7-dehydrocholesterol (7DHC, present in the skin) to previtamin D_3_ followed by the thermal isomerization to vitamin D_3_ [6]. The subsequent hydroxylation of vitamin D_3_ occurs in the liver mediated by the 25-hydroxylase (CYP2R1) which forms 25-hydroxyvitamin D_3_ (25(OH)D_3_, calcidiol), the major circulating human vitamin D metabolite. A further hydroxylation of 25(OH)D_3_ by 1-α-hydrolase (CYP27B1) in the kidney—or in extrarenal tissues such as macrophages—leads to the biologic active 1,25-dihydroxyvitamin D_3_ (1,25(OH)_2_D_3_, calcitriol) [7]. The 1,25(OH)_2_D_3_ binds with high-affinity to the VDR, which heterodimerises with the retinoid X receptor alpha (RXRα). The VDR-RXRα complex translocates into the nucleus and binds to a vitamin D response elements (VDRE) in the regulatory element region of the vitamin D target genes. Vitamin D exerts its genomic effects through the recruitment of transcriptional cofactors to this region regulating a wide variety of biological processes including calcium and phosphate absorption, cell proliferation and differentiation [8]. Approximately 2700 VDR-binding sites exist in the genome [9], explaining the wide-ranging physiologic actions of 1,25(OH)_2_D_3_. Enzymes regulate the abundance of metabolites: 24-hydroxylase (CYP24A1) limits the excess concentrations of both metabolites, [25(OH)D_3_ and 1,25(OH)_2_D_3_] by metabolic degradation. In the circulation, most vitamin D metabolites are transported to various target organs (tissues/cells) bound to a carrier protein, the vitamin D binding protein (DBP). Megalin and cubilin, two multiligand endocytic receptors, are responsible for the internalization of 25(OH)D_3_ complexed with the DBP into cells [10] (Figure 1).

The coexpression of the vitamin D system genes (e.g., *VDR* and *CYP27B1*) in multiple cell types including lymphocytes, antigen-presenting cells and pancreatic islet cells [11,12,13] highlights the importance of the vitamin D pathway in T1D.

The 25(OH)D_3_ concentration as a marker of the vitamin D status is influenced by environmental and genetic factors. Both sunlight exposure and variants in vitamin D pathway genes affect circulating 25(OH)D_3_ levels. Low 25(OH)D_3_ levels as well as specific vitamin D system gene polymorphisms enhance T1D susceptibility. Since vitamin D biosynthesis is regulated by genes, their polymorphisms (e.g., *VDR*, *CYP2R1*, *CYP27B1*, *CYP24A1*, *DBP* and *cubilin*) may alter the bioavailability as well as target effects of vitamin D metabolites.

### 2.1. Vitamin D Receptor Gene 

The VDR belongs to the nuclear receptor family of transcription factors composed of three domains: a modulating *N*-terminal dual zinc finger DNA-binding domain, a *C*-terminal ligand-binding domain and an unstructured region that links the two functional domains [14]. The human *VDR* gene spans over 100 kilobases (kb) of genomic DNA, located at chromosome 12q13.11, contains eight protein-coding exons 2–9, six untranslated exons 1a–1f, introns and 3′ UTR 1 exons [15]. Exon 1 encodes the 5′ untranslated region; exons 2 and 3 encode the DNA-binding domain, important for the interaction with the VDRE in target genes. The exons 5–9 encode the ligand-binding region responsible for 1,25(OH)_2_D_3_ binding.

Several single nucleotide polymorphisms (=SNPs; more than 5000) have been described for the human *VDR* gene (https://www.ncbi.nlm.nih.gov/snp/) and four of them have been intensively studied in relation to T1D susceptibility (Table 1).

These SNPs were identified by their restriction endonuclease cleavage sites comprising rs10735810, also known as rs2228570 (*FokI*) T → C change in exon 2, rs1544410 G → A change (*BsmI*, G = b), rs7975232 T → G change (*ApaI*, G = a), both in intron 8, and rs731236 T → C change in exon 9 (*TaqI*, T = T) [16].

The rs10735810 (*FokI*) SNP consists of a T to C substitution eliminating the first start codon (ATG) by generation of an alternative start site (ACG) leading to a differently sized protein. The shorter form (424 aa; C allele or F allele, methionine at the fourth position) is considered to be more active than the long form (427 aa; T allele or f allele, methionine at first position) [17,18]. The SNPs rs1544410 (*BsmI*), rs7975232 (*ApaI*) and rs731236 (*TaqI*) are located at the 3′ untranslated region (UTR) of the gene and are without consequences for the VDR protein structure, however, they are strongly linked to a poly(A) microsatellite repeat in the 3′ UTR. The poly(A) sequence in the 3′ UTR region of genes regulates gene expression, especially through the modulation of mRNA stability.

To date, there are 65 publications [19,20,21,22,23,24,25,26,27,28,29,30,31,32,33,34,35,36,37,38,39,40,41,42,43,44,45,46,47,48,49,50,51,52,53,54,55,56,57,58,59,60,61,62,63,64,65,66,67,68,69,70,71,72,73,74,75,76,77,78,79,80,81,82,83] on the association of *VDR* gene SNPs in T1D: these include case-control datasets [20,22,23,25,26,27,28,29,30,31,32,33,34,35,36,39,40,41,42,43,44,45,47,48,49,50,52,53,55,56,57,58,59,60,61,62,63,64,65,66,68,69,70,72,73,74], family studies [19,21,24,31,32,36,38,46,51,54,57,67,71] and meta-analyses [75,76,77,78,79,80,81,82,83] derived from several populations of different genetic background. A total of 39 publications support an association between VDR SNPs rs7975232 (*ApaI*), rs1544410 (*BsmI*), rs731236 (*TaqI*) and rs10735810 (*FokI*) alone or in combination with each other, (rs757343 *Tru9I*, rs1540339 and rs4760648) and T1D [19,21,22,23,24,26,27,28,29,30,34,35,36,37,38,40,41,42,43,44,46,48,49,50,52,53,55,56,59,60,61,62,63,65,68,69,70,71,73] (Table 2) in comparison with 16 studies that refute it [20,25,31,32,39,45,47,51,54,57,58,64,66,67,72,74].

The first study from South India examined the distribution of the VDR SNPs (rs7975232 *ApaI*, rs731236 *TaqI* and rs1544410 *BsmI*). It found a preferential transmission of the VDR “b” allele of the rs1544410 (*BsmI*) site and haplotypes “bT”, “bAT” to affected offspring [19]. In the same line, Abd-Allah et al. (2014) [68] observed in children from Egypt a significantly different rs1544410 (*BsmI*) genotype frequency between T1D and control subjects with the “b” allele the “bb” genotype conferring T1D susceptibility. Moreover, significantly more heterozygote carriers of “Aa” and “Bb” were observed in T1D patients, confirming the risk of the b allele as reported by Bonakdaran et al. (2012) [61] in an Iranian population.

In contrast, Pani et al. (2000) [21] demonstrated other VDR haplotypes “At”, “Bt” and “BAt” to confer T1D susceptibility in a German family study. Likewise, the same allele constellation “BBAAtt” was found in a cohort from South Croatia as reported by Skrabic et al. 2003 [29]. Furthermore, the association of T1D with the B allele—as risk enhancing—was confirmed in two case-control Chinese studies (Taiwanese and Han population of Beijing) [22,40], two Japanese studies [30,44] and one genetically heterogeneous Brazilian study [48]. Nevertheless, also the “AA” genotype in Taiwanese and a “t” allele in Iranian populations have been suggested as risk conferring [22,60]. Accordingly, Ramos-Lopez et al. (2006) demonstrated a higher frequency of alleles “A” and “t” within the haplotype composed of the SNPs rs9729, rs731236 (*TaqI*), rs7975232 (*ApaI*) and rs757343 (*Tru9I*) in another family study [38].

Recently, Miettinen et al. (2015) analyzed the genotype distributions of 13 VDR SNPs in a Finnish population consisting of families whose offspring had T1D (cases) and families with healthy offspring (controls) [71] where all VDR SNPs were associated with the 25(OH)D_3_ levels. Two VDR SNPs (rs1544410 *BsmI* and rs731236 *TaqI*) differed in the genotype distributions: “Bb” and “Tt” genotypes were more prevalent (corresponding to “B” and “t” allele) than in the control mothers. The investigators suggest that maternal VDR SNPs enhance a child’s risk for T1D independent of the child’s genotype. The maternal vitamin D status and VDR genotype may hereby regulate in utero development and have an influence on the later T1D risk in conjunction with environmental factors.

In addition, in Chilean T1D patients where the population is characterized by a heterogeneous admixture of people from European and South American Indian descent, a further haplotype “BAT” [43] conferred susceptibility.

Fassbender et al. confirmed the study from Mcdermott et al. (1997) with “T” as risk allele for the development of T1D [19] in a small German cohort [27], a finding later corroborated by Garcia et al. (2007) [43].

Notably, the different haplotypes associated with T1D as reported by Mcdermott et al. (1997) [19] (bAT), Pani et al. (2000) [21] (BAt) and Garcia et al. (2007) [43] (BAT) indicate a variable genetic predisposition to T1D depending on the ethnic origin. This was also shown by Audi et al. (2004) [34] by analyzing the SNP in the start codon of exon 2 (rs10735810 *FokI*) additional to the rs1544410 (*BsmI*) SNP in two Spanish populations with different genetic backgrounds (Barcelona and Navarra). A combined genotype showed that the homozygous “bbFF” genotype was more prevalent in T1D patients from Barcelona whereas the homozygous “BBFF” genotype was more frequent in Navarra. Another study conducted by San Pedro et al. (2005) included families of Basque origin where a risk-associated four-locus haplotype (fBAt) was identified [36] confirming the same profile (“BAt”) as described by Pani et al. (2000) [21]. Furthermore, haplotype analysis performed in North India showed that the haplotypes “FBAt” and “fBAT” were significantly more frequent in T1D patients [52]. Moreover, those haplotypes differed in comparison to those from South India (bAT) but were found in concordance with the “BAT” haplotype present in Chile [19,43]. In an Iranian population, the haplotypes “tAbf”, “tabF” and “tAbF” conferred an increased risk for T1D [60].

The genotype and allele distribution of rs10735810 (*FokI*) VDR SNP differs between patients and controls in many studies, however, the risk allele (F or f) also does so among the studies. The “FF” genotype and/or “F” allele predispose to T1D in Japanese, Rumanian, Uruguayan, Turkish and Iranian populations [23,24,42,55,59,61]. The “F” allele and the combination of vitamin D gene “BBFFAATt” are even considered to enhance the risk for diabetic complications, particularly diabetic nephropathy (DN) [62]. In contrast, studies from Egypt, Italy and Croatia [35,41,68] observed an association with the “ff” genotype and T1D risk. Mory et al. (2016) [73] found homozygous “ff” to be more frequent in T1D subjects with thyroid antibodies (Abs) and thyroid dysfunction in Brazil. T1D patients carrying the rs10735810 (*FokI*) SNP with thyroid peroxidase Abs showed an 18-fold risk to develop thyroid dysfunction.

Additionally, the rarely analyzed SNP rs757343 (*Tru9I*) showed an overtransmission of the allele G (corresponding to “U”) from parents to affected children as shown by Ramos-Lopez et al. 2006 and Boraska et al. 2008 [38,46]. Furthermore, in the study from Gyorffy et al. (2002) the haplotype “bau” was found more frequently in patients than in controls [26].

Furthermore, a variety of VDR allele combinations have been described as T1D protective [21,24,38,48,49,50,63,69,70].

Interestingly, one of the lowest T1D incidence rates in Europe was described for the Greek island Crete: here, two haplotypes of the four VDR SNPs confer the highest risk (aBFT and aBFt) for T1D [50]. This underscores that an interplay of genetic and environmental factors modulates T1D susceptibility.

### 2.2. Vitamin D Receptor and Meta-Analysis, Diabetes Complications and Monogenetic Vitamin D Disorders

On the basis of the diverging results of VDR SNPs and T1D susceptibility, nine meta-analyses have been performed [75,76,77,78,79,80,81,82,83]. Eight out of nine meta-analyses on the *VDR* gene and T1D published between 2006 and 2017 agree on the conclusion that rs10735810 (*FokI*) and/or rs1544410 (*BsmI*) SNPs play an important role in the development of T1D [76,77,78,79,80,81,82,83] (Table 3).

Ponsonby et al. (2008) [76] suggest that the association between the VDR SNPs and T1D should be seen as depending on the environment and not being responsible for T1D by itself. Therefore, the authors conducted a meta-analysis of 16 case-controlled studies from 19 regions and four additionally analyzed SNPs (rs7975232 *ApaI*, rs731236 *TaqI*, rs1544410 *BsmI*, and rs10735810 *FokI*) under the aspect of ambient winter UV radiation. The study centres were located across a range of latitudes from 33° S to 65° N corresponding to a winter UV radiation range from 1.0 mW/m^2^ to 133.8 mW/m^2^. The authors observed that the allele “B” of rs1544410 (*BsmI*) and the allele “F” of rs10735810 (*FokI*) were more likely risk factors for T1D under high-winter-UV radiation exposures. Four years later, Zhang et al. (2012) [77] published a meta-analysis based upon 23 case-control studies covering Asians, European and Latino populations and evaluating the ethnic-specific effects for an association with T1D. The main inclusion criteria for this meta-analysis were publications in English or Chinese; available data for genotype distributions in cases and controls; the genotype distribution of the tested controls was in Hardy–Weinberg equilibrium (HWE). Hereby, the “BB” genotype of the rs1544410 (*BsmI*) SNP was associated with an increased risk for the development of T1D, especially in Asians. This finding was also confirmed in the meta-analysis from Wang et al. 2012 [78], where 3854 cases and 6498 controls were included and an increased T1D risk for the “B” allele a particularly in East Asian population was found. In another meta-analysis, Wang et al. (2014) [79] selected 13 case-control studies (1973 T1D and 1986 controls) from the Asian region and evaluated two VDR SNPs (rs1544410 *BsmI* and rs10735810 *FokI*). Interestingly, the regional stratification analysis indicates that the rs1544410 (*BsmI*) “B” allele conferred an enhanced T1D risk in East Asia but the rs10735810 (*FokI*) allele “F” in the West Asian population. An additional meta-analysis covering the four VDR SNPs in Asian, European and Latino populations concluded that “BAT” was a significant T1D risk factor [81]. Furthermore, a recent meta-analysis on the basis of nine studies comprising 1053 children with T1D (Asian, European and Latino origin) confirmed the “BB”genotype of rs1544410 (*BsmI*) as risk marker for T1D and also for the “tt” genotype of the rs731236 (*TaqI*) SNP [83].

Liu et al. (2014) focused on the diabetes complications (diabetic nephropathy (DN) and diabetic retinopathy (DR)) and studied four variants of the VDR [82]. Hereby, the rs10735810 (*FokI*) SNP was associated with nephropathy risk in Caucasian diabetes patients, represented in a dominant model.

Apart from association studies, there are also informative case reports on genetic vitamin D disorders in T1D. One case report describes the development of T1D in a child with pre-existing hereditary vitamin D-resistent rickets (VDRR) due to a compound heterozygous mutation of the VDR (L263R and R391S) that led to dissociated responses of the CYP24A1 and RELB promoters to 1,25-Dihydroxyvitamin D_3_ action [84]. Another case of VDRR was reported from India, where a 10-year-old girl had developed T1D [85]. An additional case report with an inborn error of vitamin D metabolism was published recently. A new missense mutation of the *PHEX* gene has been described in a T1D patient from a Han Chinese pedigree over four generations that caused X-linked hypophosphatemic rickets manifesting with renal phosphate wasting, a bone mineralisation and vitamin D metabolism defect [86].

These experiments of nature underline that a vitamin D defect syndrome may have the potential for additional disease such as β-cell autoimmunity resulting in T1D. A systematic investigation of the acquired and the innate immune system in fifteen patients with VDRR showed some impairments of the innate immunity, particularly lower cathelicidin production and a proinflammatory cytokine profile of lymphocytes [87]. This illustrates the enormous plasticity of the immune system adapting to a genetic defect and that only few patients with hereditary vitamin D defect syndromes will develop an autoimmune disease such as T1D.

### 2.3. Other Vitamin D Metabolism Components

Numerous studies focused on VDR SNPs but only few on other genes of the vitamin D pathway [56,57,67,74,88,89,90,91,92,93,94,95,96,97,98,99]. Table 4 presents a summary of association studies for SNPs within the genes *CYP2R1* [57,88], *CYP27B1* [57,90,91,93], *DBP* [96,97] as well as *cubilin* [99] and T1D.

The vitamin D metabolising enzymes are all members of the cytochrome P450 superfamily of enzymes. These enzymes reside in mitochondria and contribute to the vitamin D synthesis (CYP2R1 and CYP27B1) and vitamin D degradation (CYP24A1). CYP27A1, CYP2D6, CYP2R1, CYP2C11, CYP3A4, CYP2D25 and CYP2J3 all belong to the group of hepatic cytochrome P450 enzymes with 25-hydroxylase activity. The key enzyme for the synthesis of 25(OH)D_3_ is CYP2R1 [100]. A mutation in exon 2 of the *CYP2R1* gene can abolish the 25-hydroxylase activity resulting in severe vitamin D deficiency and a rare form of rickets [101].

The *CYP2R1* gene is located on chromosome 11p15.2 with a length of 15 kb and contains five exons separated by four introns. Two SNPs (rs10741657 and rs12794714) were investigated in T1D [102]. The SNP rs10741657 (G/C) maps to a 2 kb mRNA transcript and rs12794714 (C → T, Ser → Ser) is a synonymous SNP in exon 2. Our investigations revealed that the allele G of the CYP2R1 rs10741657 SNP is more often transmitted to T1D affected offspring. Additionally, the case-control analysis shows a higher frequency of the GG genotype in T1D patients. The latter correlated with a lower 25(OH)D_3_ concentration [Ramos-Lopez et al. 2007] [88]. The subsequent analysis of a large number of samples from case/control (*n* = 8517/10,438) and T1D families (*n* = 1933) in the British population revealed an association between the two SNPs (rs10741657 and rs12794714) with T1D in a combined dataset [57].

The next enzyme in the vitamin D cascade, CYP27B1, is coded by a gene situated on chromosome 12p13.1-q13.3. The gene contains nine exons and eight introns and extends over 4.8 kb. Mutations in the *CYP27B1* gene can lead to an inactive protein unable to bind 25(OH)D_3_ as found in vitamin D dependent rickets [103]. Two SNPs within the CYP27B1 were investigated in relation to T1D, rs10877012 SNP (−1260 C/A) located in the promoter region and the rs4646536 SNP (+2838 C/T) in intron 6.

We originally observed that allele “C” and genotype “CC” were more frequent in patients with T1D than in controls [90]. Later studies, one from Egypt with 240 subjects and another one with a large collective (population different countries: British, Finland, USA, Norway, Romania) confirmed these findings [91,93]. Additionally, Bailey et al. (2007) showed also an association of T1D with the rs4646536 SNP. Cooper et al. (2011) confirmed the protective effect of the allele “A” of the rs10877012 SNP [57,91].

The last enzyme in the vitamin D cascade, CYP24A1 is capable of hydroxylating both metabolites (25(OH)D_3_, and 1,25(OH)_2_D_3_). However, the preferred substrate is 1,25(OH)_2_D_3_. CYP24A1 catabolizes 1,25(OH)_2_D_3_ in a complex of steps resulting in the production of water-soluble calcitroic acid [104]. Major alterations in the enzymatic activity of CYP24A1 can be due to mutations of the *CYP24A1* gene located on chromosome 20p13 (20.5 kb, 12 exons) that cause idiopathic infantile hypercalcemia [105]. The *CYP24A1* gene was investigated in relation to T1D susceptibility: sixteen tag SNPs for CYP24A1 that were analyzed by Bailey et al. (2007) [91] as well as two further SNPs (rs6013897 and rs2296241) did not show any association with T1D [57,89]. This gene is of potential clinical relevance because an undiagnosed *CYP24A1* mutation may cause hypercalcemia also in adults if these are exposed to high vitamin D dosages.

A further essential component of the vitamin D system is the DBP, also called group-specific component (GC) that belongs to the proteins of the albumin family and transports vitamin D in the circulation. DBP is a single chain glycoprotein with a molecular weight of 52 kDa, predominantly synthesized in the liver. The *DBP* gene maps to chromosome 4q11-q13 and contains 13 exons and 12 introns and extends over 42.5 kb. Among the many characterized DBP variants, two known SNPs in exon 11 were investigated for T1D susceptibility (rs7041 and rs4588). While the rs7041 SNP results in a T to G substitution (Aps to Glu in codon 416), rs4588 SNP leads to a C to G substitution (threonine to lysine in codon 420). The majority of the studies including the SNPs rs4588 and rs7041 and rs2282679 SNP failed to prove an association with T1D [57,67,89,94,95,98]. Two studies, however, originating from the same laboratory showed an association with rs7041 SNP and T1D [96,97]. Nevertheless, it has to be pointed out that several DBP/GC combinations of SNPs are conserved in the population forming a diverse profile of haplotypes. Such DBP haplotypes give rise to low or high affinity DBP/GC protein structures with a different binding of the free vitamin D metabolite and also affecting the monocyte production of cathelicidin [106].

Another molecule with a crucial role in vitamin D trafficking is cubilin. This 460-kDa long protein is mainly localized in the proximal renal tubule, but has been identified in other tissues including placenta, intestinal epithelium among others.

A crucial role of cubilin is the formation of an endocytic receptor complex with megalin. That complex is capable of binding DBP/25(OH)D_3_ with high affinity, mediating its uptake into the cells. The loss of functional cubilin in patients leads to loss of the 25(OH)D_3_ in urine and subsequent decrease in vitamin D metabolites plasma levels. Hence, our group examined SNPs within the *cubilin* gene as potential risk markers for T1D [99]. The *cubilin* gene is located on chromosome 10p12.33-p13. We analysed five *cubilin* SNPs (rs3740168, rs3740165, rs1801233, rs1801229 and rs2796835) in a case-control study (200 T1D and 200 controls). Out of these, the rs3740165 SNP was found to be associated with increased T1D risk. The genotype “AA” of the rs3740165 was more prevalent in T1D patients than in control but without correlation neither with 25(OH)D_3_, nor with 1,25(OH)_2_D_3_ concentration. It has to be pointed out that the SNP does not change the coding sequence in this position (Pro → Pro). Therefore, functional susceptibility may develop by a linked gene variant or a regulatory SNP.

### 2.4. Major Susceptibility to Type 1 Diabetes by HLA and Other Immune Genes: Vitamin D

The strongest susceptibility to type 1 diabetes is conferred by high risk HLA DR-DQ alleles present as hetero- or homozygous combinations most patients. There are up to 40 additional risk loci identified and some of them have been shown to affect lymphoid enhancer sequence in T and B cells, thymus and CD34^+^ stem cells [107]. Vitamin D regulates several immune genes as identified through genome wide studies by VDR chromatin immunoprecipitation followed by mass DNA sequencing (CHIP-seq) [108,109] where VDR binding to autoimmune susceptibility loci was identified amongst them type 1 diabetes sites. Hereby, the VDR-enhanced susceptibility to T1D may form a genetically determined proinflammatory cytokine pattern [110].

### 2.5. Vitamin D Intervention in Type 1 Diabetes and Pharmacogenomics

Vitamin D deficiency is a worldwide problem [111]. It enhances the risk for various conditions including T1D [112] and provides the rationale for many intervention trials (clincaltrials.gov currently—as of 11 April 2017—3122 trials listed). The potential to modify the development of T1D was reported in a case-control study and a birth cohort follow-up study from Finland: it strongly indicated that vitamin D supplementation in infancy decreases the risk of T1D [113,114]. The therapeutic benefit of vitamin D onT1D was tested in some clinical trials [2] but only few studies examined the effect of the vitamin D SNPs in the context of vitamin D supplementation for T1D [115,116,117]. We recently performed a randomized, double-blind, placebo-controlled trial with cross-over design in which thirty-nine patients with T1D received 4000 IU/day cholecalciferol for three months followed by placebo or in reverse sequence. Hereby, besides an improvement of the vitamin D status (median 25(OH)D_3_ increased to 38 ng/mL), the regulatory T cells (Treg) demonstrated a differential response to vitamin D to three months’ treatment according to VDR SNPs. Furthermore, this trial also showed an improvement of glycemic parameters under vitamin D treatment. Patients carrying the genotypes aa, TT and bb (rs7975232 *ApaI*, rs731236 *TaqI* and rs1544410 *BsmI*) were capable of raising their Treg cell number [115]. A further study tested in vitro the functional role of the VDR rs10735810 (*FokI*) SNP in T-helper (CD3^+^CD4^+^) from twenty patients with T1D. The stimulation of CD3^+^CD4^+^ cells with 25(OH)D_3_ [62.4 nM] and 1,25(OH)_2_D_3_ [1 × 10^−8^ M] for 72 h revealed a reduced percentage of CD4^+^ cells isolated from T1D patient carrying “FF”, suggesting a beneficial balance in the T cell compartment [116].

In a prior in vitro study, Mauf et al. (2015) [117] had explored the immunomodulatory effects of vitamin D supplementation on 25(OH)D_3_ levels, on dendritic cells in twelve patients with T1D and the role of the vitamin D SNPs. Remarkably, the 25(OH)D_3_ treatment (50 ng/mL) for seven days inhibited the differentiation of monocytes into dendritic cells, promoting the formation of intermediate cells (IC). The increase of IC under supplementation with 25(OH)D_3_ was related to the genotypes of two *VDR* SNPs (rs731236 *TaqI* and rs1544410 *BsmI*) and one SNP of the *CYP24A1* gene (rs927650), illustrating that the immune effects of vitamin D supplementation can depend on genetic variants of the vitamin D system.

## 3. Conclusions

Vitamin D deficiency is a risk factor for T1D and genes of the vitamin D system show robust associations with T1D. The vitamin D system appears to affect the immune regulatory pathways, leading to the final β-cell destruction. Several experimental lines of evidence suggest islet protection by vitamin D. Exploiting this potential will be a challenge for future studies, including larger controlled trials with different doses of vitamin D and functional studies to elucidate mechanistic actions in the immune system and also for metabolic end points.

## Figures and Tables

**Table 1:** Type 1 diabetes (T1D) and vitamin D pathway associated single nucleotide polymorphisms (SNPs).

Acronym	Full Name	Protein Function	Chr	Position	SNP	locus	Gene Function	Amino Acid Change
*VDR*	vitamin D receptor	transcription factor transcriptional control of vitamin D target genes	12	12q13.11	rs7975232	intron 8		no
					rs10735810	exon 2	missense	Met → Thr
					rs1544410	intron 8		no
					rs731236	exon 9	synonymous	Ile → Ile
*CYP2R1*	vitamin D 25-hydroxylase	transforming photo- synthesized and dietary vitamin D into 25(OH)D_3_	11	11p15.2	rs10741657	5′ near gene	2 kb mRNA transcript	
					rs12794714	exon 1	synonymous	Ser → Ser
*CYP27B1*	25(OH)D 1-α-hydroxylase	conversion of 25(OH)D_3_ to 1,25(OH)_2_D_3_	12	12q14.1	rs10877012	5′ near gene	promoter (−1260)	
					rs4646536	intron 6	(+2838)	no
*DBP or GC*	vitamin D binding protein or group-specific component	transport of vitamin D metabolites	4	4q11.13	rs4588	exon 11	missense	Thr → Lys
					rs7041		missense	Asp → Glu
*CUBN*	cubilin	endocytotic receptors capable to internalize vitamin D into the cells	10	10p12.33-p13	rs3740165		synonymous	Pro → Pro

rs7975232 (=*ApaI*), rs10735810 (=*FokI*), rs1544410 (=*BsmI*) and rs731236 (=*TaqI*).

**Table 2:** T1D and summary of association studies for VDR SNPs. Diabetic retinopathy (DR); diabetic nephropathy (DN); *Staphylococcus aureus* carriage (SAC); antibodies (Abs).

*VDR* Gene								Susceptibility to T1D SNPs	
	Reference	Author	Year	Population	Total	Case	Control		Comparison Groups
1	[19]	McDermott et al.	1997	Indian	93			rs1544410, bt, bAT	T1D families
2	[21]	Pani et al.	2000	German	152			At, Bt, Bat	T1D families
3	[22]	Chang et al.	2000	Chinese (Taiwan)	405	157	248	rs7975232, rs1544410	T1D/control
4	[23]	Ban et al.	2001	Japanese	360	110	250	rs10735810	T1D/control
5	[24]	Guja et al.	2002	Romanian	204			rs10735810, rs731236	T1D families
6	[26]	Gyorffy et al.	2002	Hungarian	210	107	103	bau	T1D/control
7	[27]	Fassbender et al.	2002	German	132	75	57	rs731236	T1D/control
8	[28]	Taverna et al.	2002	French	200	101	99	rs731236	T1D with/without DR
9	[29]	Skrabic et al.	2003	Croatian (Dalmatian)	266	134	132	BBAAtt	T1D/control
10	[30]	Motohashi et al.	2003	Japanese	425	203	222	rs1544410	T1D/control
11	[34]	Audi et al.	2004	Spanish (Barcelona)	429	155	274	rs1544410, rs10735810, bbFF	T1D/control
				Spanish (Navarre)	205	89	116	rs1544410, rs10735810, bbff	T1D/control
12	[35]	Zemunik et al.	2005	Croatian (Dalmatian)	266	134	132	rs10735810, FbATU	T1D/control
13	[36]	San Pedro et al.	2005	Spanish (Basque)	159	71	88	fBAt	T1D/control
						136	119		T1D families
14	[37]	Taverna et al.	2005	French	254	126	128	rs10735810	T1D with/without DR
15	[38]	Ramos-Lopez et al.	2006	German	254			rs9729, rs731236, rs7975232, rs757343	T1D families
16	[40]	Xiao et al.	2006	Chinese		54	82	rs1544410	T1D/control
17	[41]	Capoluongo et al.	2006	Italian		246	246	rs10735810	T1D/control
18	[42]	Mimbacas et al.	2007	Uruguayan	45			rs10735810	T1D families
19	[43]	Garcia et al.	2007	Chilean	419	216	203	BAT	T1D/control
20	[44]	Shimada et al.	2008	Japanese	1373	774	599	rs1544410	T1D/control
21	[46]	Boraska et al.	2008	Croatian	160			rs757343, rs757343-rs1544410	T1D families
22	[48]	Mory et al.	2009	Brazilian	383	189	194	rs1544410	T1D/control
23	[49]	Panierakis et al.	2009	Greece	93	29	64	rs7975232, rs731236	T1D with/without SAC
24	[50]	Panierakis et al.	2009	Greece	196	100	96	rs7975232, rs731236, rs1544410, rs10735810	T1D/control
25	[52]	Israni et al.	2009	Indian	424	233	191	FBAt, fBAT	T1D/control
26	[53]	Bucan et al.	2009	Croatian	120	66	54	rs1544410	T1D with/without DR
27	[55]	Kocabas et al.	2010	Turkish	176	90	86	rs10735810	T1D/control
28	[56]	Martin et al.	2010	UK, Irish	1329	655	674	AGT	T1D with/without DN
29	[59]	Sahin et al.	2012	Turkish	165	85	80	rs10735810	T1D/control
30	[60]	Mohammadnejad et al.	2012	Iranian	187	87	100	rs731236, tAbf, tabF, tAbF	T1D/control
31	[61]	Bonakdaran et al.	2012	Iranian	114	69	45	rs7975232, rs1544410, rs10735810	T1D/control
32	[62]	Vedralová et al.	2012	Czech	172	54	118	rs10735810	T1D/control
					250	132	118	rs10735810, BBFFAATt	DN/control
33	[63]	Frederiksen et al.	2013	North American		38	84	rs1544410	T1D+IA/IA
34	[65]	De Azevedo et al.	2013	Brazilian	421	204	217	rs1540339, rs4760648	T1D/control
35	[68]	Abd-Allah et al.	2014	Egyptian	240	120	120	rs1544410, rs10735810	T1D/control
36	[69]	Kamel et al.	2014	Egyptian	102	74	28	rs7975232, rs731236	T1D/control
37	[70]	Cheon et al.	2015	Korean	194	81	113	rs731236, rs1544410	T1D/control
38	[71]	Miettinen et al.	2015	Finnish	2854			rs731236, rs1544410	T1D families
39	[73]	Mory et al.	2016	Brazilian		180		rs10735810	T1D with/without Abs

rs7975232 (=*ApaI*), rs10735810 (=*FokI*), rs1544410 (=*BsmI*), rs731236 (=*TaqI*) and rs757343 (=*Tru9I*).

**Table 3:** Meta-analysis of VDR SNPs and T1D and diabetic nephropathy (DN).

*VDR* Gene and Meta-Analysis								Susceptibility to T1D SNPs	
	Reference	Author	Year	Population	Total	Case	Control		Comparison Groups
1	[76]	Ponsonby et al.	2008	Asian, European, Latinos	18,257	2549	15,708	rs1544410, rs10735810	T1D/control
		16 studies							
2	[77]	Zhang et al.	2012	Asian, European, Latinos	11,591	5335	6256	rs1544410	T1D/control
		26 studies							
3	[78]	Wang et al.	2012	East Asian	10,352	3854	6498	rs1544410	T1D/control
		25 studies							
4	[79]	Wang et al.	2014	East Asian, West Asian	3959	1973	1986	rs1544410, rs10735810	T1D/control
		13 studies							
5	[81]	Tizaoui et al.	2014	Asian, European, Latinos	8753	3332	5421	BAT, bAT	T1D/control
		26 studies							
6	[82]	Liu et al.	2014	French, Polish, Croatian, Irish, Czech Iranian, Chinese	2734	1394	1340	rs10735810	diabetic + DN/control
		8 studies							
7	[83]	Sahin et al.	2017	Asian, European, Latinos	2070	1053	1017	rs1544410, rs731236	T1D/control
		8 studies							

The meta-analysis published by Qin et al. [80] (23 studies, Asian, Latino, African and Caucasian) is not included in the Table because only an abstract was available. “B” allele of the rs154410 (=*BsmI*) SNP was associated with an increased risk for the development of T1D especially in Asians. rs10735810 (=*FokI*) and rs731236 (=*TaqI*).

**Table 4:** T1D and a summary of association studies for SNPs within the genes *CYP2R1*, *CYP27B1*, *DBP* and *cubilin*.

Other Vitamin D System Components						Susceptibility to T1D SNPs	
Author	Year	Population	Total	Case	Control		Comparison Groups
***CYP2R1* gene**							
Ramos-Lopez et al. [88]	2007	German	203			rs10741657	T1D families
			578	284	294	rs10741657	T1D/control
Cooper et al. [57]	2011	British	1933			rs10741657, rs12794714	T1D families
			18,955	8517	10,438	rs10741657, rs12794714	T1D/control
***CYP27B1* gene**							
Ramos-Lopez et al. [90]	2004	German	572	252	320	rs10877012	T1D/control
Bailey et al. [91]	2007	Great Britain, Northern Ireland,	2774			rs10877012, rs4646536	T1D families
		Finland, USA, Norway, Romania					
		Great Britain	16,612	7854	8758	rs10877012, rs4646536	T1D/control
Cooper et al. [57]	2011	British	1933			rs10877012	T1D families
			18,955	8517	10,438	rs10877012	T1D/control
Hussei et al. [93]	2012	Egyptian	240	120	120	rs10877012	T1D/control
***DBP (GC)* gene**							
Ongagna et al. [96]	2001	Alsatian and North African origin	95	43	52	rs7041	T1D/control
Ongagna et al. [97]	2005		178	110	68	rs7041	T1D/control
***Cubilin* gene**							
Ramos-Lopez et al. [99]	2010	German	400	200	200	rs3740165	T1D/control
